# Book Review: Evolution of the Learning Brain: Or How You Got to Be So Smart…

**DOI:** 10.3389/fpsyg.2020.00100

**Published:** 2020-02-04

**Authors:** Majid Manoochehri, Kazem Nematollahzadeh Mahani

**Affiliations:** ^1^Department of Psychology, Islamic Azad University, Kerman, Iran; ^2^Department of Psychology, Islamic Azad University, Zarand, Iran

**Keywords:** evolution of learning, learning system, education, cultural evolution, language

In his new book, Howard-Jones (2018) a professor of neuroscience at the University of Bristol, provides the reader with an overview of the evolution of species, and throughout the book tries to find the ways in which evolution changes nervous systems and affects learning abilities.

He commences with a brief introduction of the evolution theory in the first two chapters and then dives into profound discussions. While equipped with diverse learning systems, vertebrates, including humans, do not have to switch off one system before turning on another. Instead, two, three or all their learning systems can be used together. Even the simplest of learning situations will probably need more than one system and these systems have evolved to interact in most everyday learning tasks (Chapter 3). This brain's ability of vertebrates is highlighted by Howard-Jones and considered as a support for the limited capacity of working memory (Cowan, 2001).

Howard-Jones highlights the fact of increasing the size of the primates' brain during the evolution and in the light of the *social brain hypothesis* (Dunbar, 1998) notes that the reason appears to be the increasingly social nature of their niche (Chapter 4). Intragroup competitions, maintaining group cohesion, and social learning have been discussed elsewhere (Di Paolo et al., 2018) to drive the evolution of social intelligence, but the one which is the major interest here, is social learning. According to Howard-Jones, what distinguishes primates from other animals is their interest and ability to understand events they are not directly involved in, though there are of course other close views on this, such as “the level of information obtained through learning” (Larsen, 2010). However, this characteristic is one of the cornerstones of Howard-Jones' discussion.

Given the expense of neural tissue in terms of energy, an important question, is the advantage of having a bigger brain (Chapter 5). Contrary to other possibilities, such as tool-use (Ambrose, 2001), Howard-Jones discusses that it was social factors, such as “cooperative breeding” or “social learning” that drove brain expansion. He discusses that intelligence can be affected by both biological inheritance and environmental influences and this genetic–environmental feedback loop boosted the rewards of cerebral expansion and made genetic changes more impactful.

Regarding the emergence of culture and language (Chapter 6), he discusses how creating gestures in a cooperative context helped spoken language develop in prehistory. Abstract symbols, because of their particular features encourage learning. Symbolic language allows for more elaborate and more efficient communication, and so the possibility of faster learning. Besides, when we turn concrete experience into abstract symbols, it becomes easier and faster to mentally manipulate information and generate thoughts. Emerging language when environmental conditions were good, allowed communities to connect efficiently and eventually caused the cultural explosions. Although, compelling evidence is provided to support this argument, some other data are not considered, such as Coolidge and Wynn (2005) hypothesis, suggesting that a genetic mutation caused modern thinking and cultural explosion.

In the present time human's brain is special enough for numeracy and literacy to emerge through learning, rather than evolution (Chapters 7, 8). Howard-Jones discusses that although some pre-existing facilities, such as “subitizing” ability—the ability to recognize four objects at once without counting—or the hands and fingers have supported the emergence of numeracy, we have noticeable limits and we did not evolve for some areas of learning such as numeracy, literacy, or formal learning in universities. In fact, the demands of our progressive culture have advanced beyond our evolved genetic abilities. He stipulates that tools, such as numeracy and literacy, use our old biology in new ways. From his view, the creation of writing appears to be a response to life getting complicated, when human communities began to urbanize.

The upshot of the book can be found in Chapter 9; a better understanding of our evolutionary background results in discovering numerous elements that have the potential of influencing learning or can be used to improve education. Some factors have been suggested and discussed to have this potential, such as “attracting students' attention according to our evolutionary tendencies,” “testing,” “sleep,” and “enactment effect,” though some other possibilities are not considered or broadly discussed, such as the importance and application of different types of associative and non-associative learning (Dugatkin, 2013). However, Howard-Jones' major goal seems to be not to merely concentrate on providing practical methods, which other neuroeducational resources broadly concentrated on (e.g., Clabough, 2019), but rather to set the theoretical basis for the future research.

Chapter 10 is certainly the most exciting part of this journey with Jones; at the point, he tries to predict the future of the learning brain and humanity. Far from the notion that the human evolution is over by the present time, proposed by some scientists, such as Steve Jones or Ian Tattersall (Owen, 2009), Howard-Jones presents and discusses different future scenarios from extinction to enhancement.

Using a sophisticated combination of biology, neuroscience and educational sciences, which is the distinctive characteristic and the advantage of the book over similar works, this publication provides novel insights and a better understanding of the learning brain. One thing to like about this book is its critical look at terms of “disorder” and “abnormal” (e.g., Dyslexia, ADHD). It takes them as a genetic variation that has developed over deep time and been unproblematic until the present time. The last chapter of this book—predicting the future of cognition and humanity based on the available evolutionary knowledge—is in fact the missing piece of contemporary psychological discussions.

On the other side, the author does not go through the details in some arguments, which may lead to ignoring some determinant aspects. Besides, attempts to edit our own genome in order to produce an improved version of our species, will primarily target our “genetic variation,” the danger of which is underestimated here (last chapter). However, in summary, this is a worthwhile contribution to the psychology literature, one that readers will enjoy and one that will stimulate many new research ideas.

## Author Contributions

MM provided the first draft. KM revised the manuscript. All authors approved the final version.

### Conflict of Interest

The authors declare that the research was conducted in the absence of any commercial or financial relationships that could be construed as a potential conflict of interest.
